# Light-Elicited and Oxygen-Saved Iridium Nanocapsule for Oxidative Damage Intensified Oncotherapy

**DOI:** 10.3390/molecules28114397

**Published:** 2023-05-28

**Authors:** Guobo Chen, Xiang Wang, Zongyan He, Xueyu Li, Zhijin Yang, Yule Zhang, Yuhao Li, Lulu Zheng, Yuqing Miao, Dawei Zhang

**Affiliations:** 1 School of Materials and Chemistry, Institute of Bismuth, University of Shanghai for Science and Technology, Shanghai 200093, Chinayqmiao@usst.edu.cn (Y.M.); 2 Shanghai Environmental Biosafety Instruments and Equipment Engineering Technology Research Center, Engineering Research Center of Optical Instrument and System, The Ministry of Education & Shanghai Key Laboratory of Modern Optical System, University of Shanghai for Science and Technology, Shanghai 200093, China; 15195241112@163.com (Z.Y.);

**Keywords:** iridium complex, TH287, self-assembly, enhanced photodynamic therapy, oxidative damage

## Abstract

Regulating redox homeostasis in tumor cells and exploiting oxidative stress to damage tumors is an efficacious strategy for cancer therapy. However, the strengths of organic nanomaterials within this strategy are often ignored. In this work, a light-triggered reactive oxygen species (ROS) damaging nanoamplifier (IrP-T) was developed for enhanced photodynamic therapy (PDT). The IrP-T was fabricated with an amphiphilic iridium complex and a MTH1 inhibitor (TH287). Under green light stimulation, IrP-T catalyzed the oxygen in cells to generate ROS for realizing oxidative damage; meanwhile, TH287 increased the accumulation of 8-oxo-dGTP, further strengthening oxidative stress and inducing cell death. IrP-T could maximize the use of a small amount of oxygen, thus further boosting the efficacy of PDT in hypoxic tumors. The construction of nanocapsules provided a valuable therapeutic strategy for oxidative damage and synergizing PDT.

## 1. Introduction

Photodynamic therapy (PDT) is a promising technology based on photosensitizers, light sources, and endogenous oxygen that enables temporal and spatial control of photosensitizer activation [1,2,3,4]. When the photosensitizer absorbs enough light to reach the singlet excited state, the molecules in that state can cross over to the triplet excited state via an intersystem crossing and triplet excited states have longer lifetimes than singlet excited states [5,6,7]. After the photosensitizer reaches a triplet excited state, its energy can return to the ground state via radiation and non-radiation transitions and react with oxygen or endogenous molecules in cells to produce reactive oxygen species (ROS) [3,8]. In the periodic table of elements, iridium is a transition metal element [9]. Because of its higher atomic number, iridium induces stronger spin–orbit coupling and increases intersystem crossing, making iridium complexes a potential photosensitizer for PDT [10,11,12,13].

The redox imbalance of tumor cells will amplify oxidative stress [14,15]. To reduce oxidative stress damage, tumor cells have developed a variety of self-repair mechanisms [16,17]. Among them, MutT homolog1 (MTH1) in tumor cells plays a crucial role in DNA replication [18]. MTH1 is a MutT homologous enzyme, which can convert 8-oxo-2′-deoxyguanosine triphosphate (8-oxo-dGTP) to 8-oxo-2′-deoxyguanosine monophosphate (8-oxo-dGMP) preventing ROS-induced oxidative DNA damage [19,20,21,22,23]. Thus, ROS accumulation for tumor therapy can be effectively improved by preventing this self-repair mechanism of tumor cells. The small molecule MTH1 protein inhibitors most widely used today are two small molecules, TH287 and TH588. Both of them can effectively inhibit MTH1 protein expression in cancer cells. The maximum half-inhibitory concentration of the cells is 0.8 ± 0.1 nM and 5.0 ± 0.2 nM, respectively [24,25,26,27,28].

**Scheme 1:**
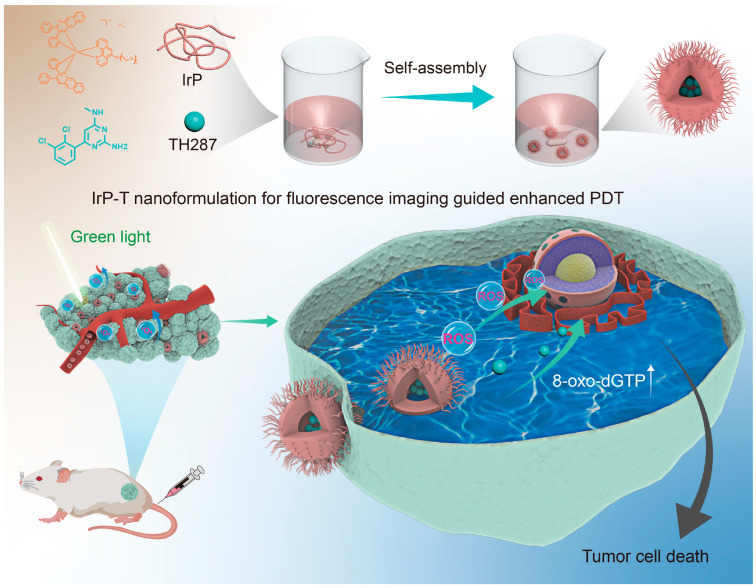
Schematic diagram of IrP-T for tumor-enhanced photodynamic therapy including self-assembly diagram of IrP-T and its theranostic nanoplatform for oncotherapy.

Using nanomaterials as carriers to deliver drugs to tumor sites can currently improve drug utilization [29,30]. In this study, we designed an amphiphilic iridium complex (IrP) to construct an optical imaging-guided enhanced photodynamic therapy (EPDT) theranostic nanoplatform by self-assembly (Scheme 1). TH287 (hereinafter T) was loaded in IrP using a solvent-assisted method to form nanomicelles (IrP-T). Under light excitation, IrP-T can react with intracellular oxygen to generate singlet oxygen (^1^O_2_), which causes the imbalance of the cell redox state and increases the content of 8-oxo-dGTP. Simultaneously, the intracellular release of TH287 can prevent the hydrolysis of 8-oxo-dGTP, thereby blocking oxidative damage repair and sending cells into irreversible programmed death. This design not only ameliorates the poor water solubility of the iridium complex and TH287 but also further enhances the PDT effect via the synergy of PDT and the oxidative damage repair blocking strategy.

## 2. Results and Discussion

### 2.1. Preparation and Characterization of IrP and IrP-T

The synthetic route of the amphiphilic iridium complex IrP was depicted in Scheme S1. Firstly, dpqb was synthesized by a Schiff base reaction, and then the intermediate [Ir(dpqb)_2_]_2_Cl_2_ was synthesized by the reaction of dpqb with IrCl_3_ according to the principle that Ir^3+^ can coordinate with the C^N ligand. Following that, the polyethylene glycol-modified N^N ligand was synthesized, and Phen-mPEG2000 was obtained via a substitution reaction of 5-bromo-1,10-phenanthroline with polyethylene glycol monomethyl ether. Under the catalysis of CF_3_SO_3_Ag, iridium in the bridged chloride complex can coordinate with the N^N ligand of Phen-mPEG2000 to form the amphiphilic iridium complex IrP. The final product was characterized by ^1^H-NMR and high-resolution mass spectrometry (Figure S1), and all characterizations were recorded during the synthesis step. IrP has good solubility in organic solvents. In addition, with the help of polyethylene glycol, the solubility of IrP in water has also been greatly improved, which makes IrP an amphiphilic complex.

**Figure 1:**
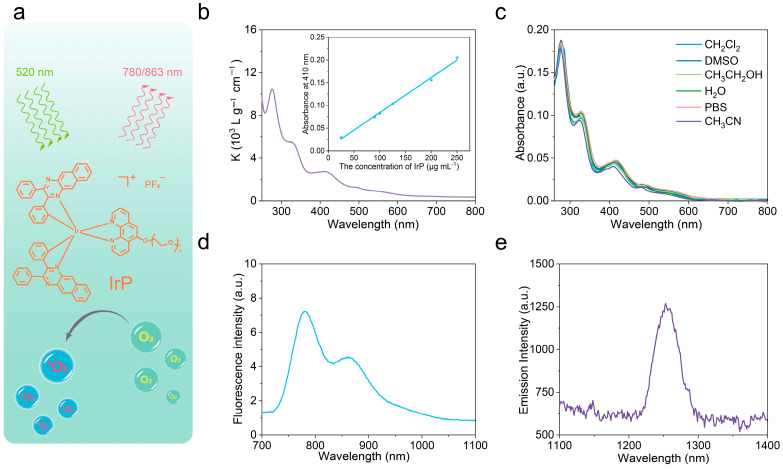
Photophysical characteristics of IrP. (**a**) Schematic illustration of IrP with the characteristics of NIR emission and ROS production; (**b**) Absorption spectra of the mass extinction coefficient of IrP in water. (Inset: absorption of IrP at 410 nm with concentration and its linear fitting curve); (**c**) Absorption spectra of IrP in different solvents; (**d**) Emission spectra of IrP in dichloromethane (λ_ex_ = 520 nm); (**e**) ^1^O_2_ emission peak of IrP (λ_ex_ = 420 nm).

It is well known that iridium complexes have large Stokes shifts, high quantum yields, and tunable absorptions and emissions, allowing them to be applied in PDT and optical imaging (Figure 1a) [31,32,33,34]. The photophysical properties of IrP were investigated first. Figure 1b showed that IrP has favorable light absorption properties in both the ultraviolet and visible ranges. The embedding diagram displayed that the concentration of IrP is linearly related to its optical absorption, which conforms to the Beer–Lambert Law, indicating that IrP does not aggregate with increasing concentrations in an aqueous solution and has advantageous water solubility. As the concentration of TH287 increased, so did the absorption peak of IrP-T at 280 nm. Meanwhile, the embedding diagram showed that the TH287 concentration had a linear relationship with the light absorption intensity at 280 nm, which was consistent with the Beer–Lambert Law (Figure S2). It could also be demonstrated that TH287 did not aggregate in an aqueous solution and could be loaded successfully in IrP. In addition, the absorption of IrP did not change with the solvent, confirming that IrP had excellent stability (Figure 1c). The room temperature emission spectrum of IrP was investigated because iridium has a high atomic number, which is propitious to the phosphorescence of iridium complexes. IrP has an obvious near-infrared (NIR) window emission at 780 nm and 863 nm when excited by 520 nm light, enabling it to be used for in vivo imaging (Figure 1d). The remarkable light absorption ability, large Stokes shift, and fantastic stability means IrP has potential in tumor therapy and imaging.

There are two mechanisms for photosensitizers to produce ROS after being exposed to light, so the species of the produced ROS are different. The ROS species produced through the Type I pathway are •O_2_^−^ and •OH, while the ROS produced through the Type II pathway is ^1^O_2_. To begin, DPBF was used as an ROS-detection agent to confirm the type of ROS generated by IrP [35]. Under 520 nm excitation, the degradation rate of DPBF increased as the IrP concentration increased. When fixing the IrP concentration, the degradation rate of DPBF accelerated with the increase in laser power, denoting that the IrP concentration and laser power are positively correlated to ROS production (Figure S3). Since DPBF can simultaneously capture •O_2_^−^ and ^1^O_2_, nitrotetrazolium blue chloride (NBT) was used to further verify whether IrP produces •O_2_^−^ under light excitation. Under 520 nm light irradiation, the absorption peak of the mixture of NBT and IrP at 260 nm did not change with the extension of the illumination time, indicating that there was no •O_2_^−^ generation (Figure S4). Then, the ROS produced by IrP was further proved to be ^1^O_2_ by a fluorescence spectroscopy. Since the energy of the excited oxygen molecule was higher than that of the ground state, when the electron transitioned from the triplet excited state back to the ground state, it released energy in the form of light and emitted phosphorescence. There are typically two methods for ^1^O_2_ transition: ^1^O_2_ → O_2_ + hv (1268 nm); 2^1^O_2_ → 2O_2_ + hv (634 nm and 703 nm). When IrP was excited, there was a clear emission peak at around 1268 nm that can be attributed to the emission of ^1^O_2_, indicating its generation (Figure 1e). Therefore, the ROS was generated by IrP through the Type II mechanism and the ROS type was ^1^O_2_.

**Figure 2:**
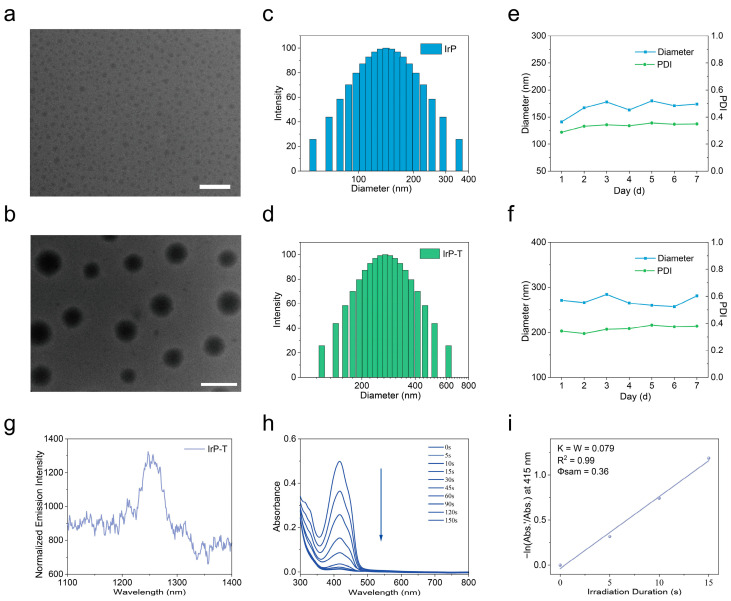
Structural characterization and ROS identification of IrP and IrP-T. TEM images of IrP (**a**) and IrP-T (**b**); DLS diagram of IrP (**c**) and IrP-T (**d**); Stability of IrP (**e**) and IrP-T (**f**) in water; (**g**) ^1^O_2_ emission of IrP-T; (**h**) Degradation curve of the mixture of IrP-T and DPBF with time under 520 nm light excitation; (**i**) Calculation of ^1^O_2_ yield of IrP-T. (Scale bar: 500 nm).

Simultaneously, the addition of TH287 during the self-assembly process can cause it to be loaded into the cavity of IrP nanomicelles to obtain IrP-T nanomicelles. The morphology and size of IrP and IrP-T were characterized by TEM. As shown in Figure 2b, both IrP and IrP-T are spherical nanoparticles with average diameters of 83 nm and 260 nm, respectively (Figure S5). The particle size of IrP-T is larger than that of IrP, possibly because TH287 was loaded into the inner cavity of IrP to form larger nanoparticles. The hydrated particle sizes of IrP and IrP-T were measured by a DLS analyzer, which were 141 nm and 271 nm, respectively (Figure 2c,d). The polyethylene glycol on the nanomicelle surface combined with water molecules to form a hydration layer, which may explain why the hydrated particle size was larger than the TEM statistical particle size. Accordingly, the hydration particle size and polymer dispersity index (PDI) of IrP and IrP-T were measured by DLS for seven days. The numerical fluctuations of IrP and IrP-T were not noticeable, demonstrating that IrP and IrP-T were stable in an aqueous solution (Figure 2e,f). In addition, for photosensitizers, the ^1^O_2_ yield most intuitively reflected their in vitro photodynamic effect. As shown in Figure 2g–i, the ROS produced by IrP-T under 520 nm light excitation was determined to be ^1^O_2_ and the ROS generation yield was 0.36, indicating that IrP-T also had a respectable ^1^O_2_ yield, laying the groundwork for the synergistic oncotherapy strategy of PDT and oxidative damage repair blocking.

### 2.2. In Vitro EPDT Effect Evaluation

**Figure 3:**
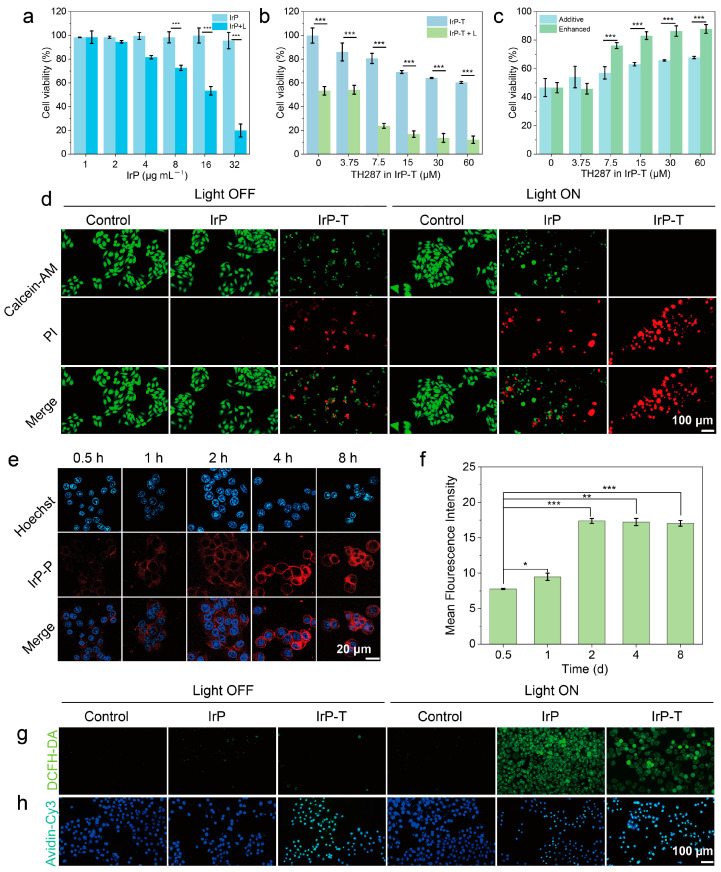
Evaluation of EPDT effect in vitro. (**a**) Cell viability with or without laser irradiation after co-incubation of 4T1 cells with IrP; (**b**) Cell viability with or without laser irradiation after co-incubation of 4T1 cells with IrP-T; (**c**) Enhanced PDT (IrP-T + L) and theoretical addictive therapy (IrP + L and IrP-T); (**d**) Living cells (green) and dead cells (red) evaluation with Calcein-AM and PI staining after different treatments; (**e**) The cells were incubated with IrP-P (red) for 0.5 h, 1 h, 2 h, 4 h, and 8 h, respectively, then used hoechst (blue) to localize the nucleus, and photographed with a confocal microscope (λ_ex_ = 488 nm, λ_em_ = 630 nm); (**f**) A corresponding statistical chart of average fluorescence intensity; (**g**) ROS detection in cells from the fluorescence of DCF (green) with DCFH-DA staining after different treatments; (**h**) Detection of intracellular 8-oxo-dGTP after different treatments and used hoechst (blue) to localize the nucleus. (* *p* < 0.05, ** *p* < 0.01, and *** *p* < 0.001).

In vitro photodynamic effect experiments showed that IrP could continuously produce ^1^O_2_ under 520 nm laser irradiation. In order to investigate the therapeutic effect of IrP-T in vitro, a CCK-8 assay was used to evaluate the proliferation inhibition effect of PDT on 4T1 cells. IrP was co-incubated with cells for 24 h in the dark. Even as the concentration of IrP increased to 32 μg mL^−1^, cell viability remained above 90%, indicating that IrP did not cause significant toxicity to cells in the absence of light stimulation. However, under the irradiation of 520 nm light (100 mW cm^−2^), cell viability decreased with an increasing IrP concentration. Specifically, IrP reduced the proliferation ability of cells by about 80% after light irradiation at 32 µg mL^−1^ concentration (Figure 3a). The IC_50_ value of PDT was calculated to be 15.68 µg mL^−1^ (Figure S6), and the phototoxicity index (defined as the ratio of IC_50_ values of dark toxicity to the phototoxicity of the material) was greater than 100 [11]. Based on this, the therapeutic effect of IrP-T was then assessed. We fixed the IrP concentration at 16 µg mL^−1^, as seen in Figure 3b, and the cell viability of IrP-T gradually decreased as the concentration of TH287 increased. However, high concentrations of TH287 did not inhibit cell viability significantly, probably because the inhibition of MTH1 in mouse breast cancer cells reached a threshold. In contrast, cell proliferation was remarkably inhibited when IrP-T was irradiated with 520 nm light, and the cell viability inhibition rate was more than 80% when the concentration of TH287 reached 15 µM. Compared with pure PDT, EPDT is more effective. To evaluate the EPDT effect, the following formula was used for quantitative analysis [36]:

$$T_{Additive}=100-\left(f1\times f2\right)\times 100$$

$$T_{Enhanced}=100-f_{Enhanced}$$

Here, f1 and f2 represent the percentage of cell survival after IrP + L and IrP-T treatment, respectively; $f_{Enhanced}$ represents the percentage of cell survival after IrP-T + L treatment. $T_{Additive}$ and $T_{Enhanced}$ represent the theoretical cell inhibition rates and the actual cell inhibition rates after the two treatments, respectively. Figure 3c showed that as TH287 concentration increased, the cell inhibition rate of additive therapy increased slowly, whereas the inhibition rate of enhanced therapy increased rapidly. Moreover, when the concentration of TH287 reached 15 µM, the cell inhibition rate exceeded 80%, indicating that synergistic therapy has a favorable EPDT effect.

Subsequently, a live/dead cells fluorescence staining kit (Calcein-AM/PI) was further applied to validate PDT-induced apoptosis. Calecin-AM itself has no fluorescence, but it can enter the living cells to react with the enzyme to produce Calecin with green fluorescence, while PI can enter the dead cells to produce red fluorescence (Figure 3d). The IrP-T + L group exhibited the strongest red light with the weakest green light, indicating that the EPDT caused the most cell death, which was consistent with the CCK-8 results, further consolidating the synergistic treatment effect.

As a drug-loaded nanoplatform for diagnosis and treatment, the therapeutic effect of IrP is first evaluated by cell uptake. Since the emission of IrP is beyond the collection range of the confocal fluorescence microscope, we wrapped the red light-emitting dye PpIX in IrP to form IrP-P to observe the uptake of nanomicelles by cells. IrP-P was incubated with 4T1 cells, and cell uptake was photographed at 0.5, 1, 2, 4, and 8 h. As shown in Figure 3e, red fluorescence was observed at 0.5 h, indicating that IrP-P could be taken up quickly by the cells. The red fluorescence became more visible as the co-incubation time was prolonged, demonstrating that the cell uptake of IrP-P increased. A quantitative analysis of fluorescence intensity showed that the fluorescence intensity of IrP-P increased rapidly within 2 h of incubation (Figure 3f). This proves that IrP, as a drug-loaded nanoplatform, can be effectively uptaken by cells, allowing for timely drug efficacy.

Photodynamic performance was then further evaluated in cells by measuring ROS production. An ROS fluorescent detection probe (DCFH-DA) was utilized to assess intracellular ROS levels. DCFH-DA itself does not have luminescent properties; however, when DCFH-DA enters the cells, it can react with high levels of intracellular ROS to produce DCF, which can emit green light under light excitation at around 488 nm. As shown in Figure 3g, cells incubated with IrP and IrP-T showed no green fluorescence without external light stimulation, signifying that IrP and IrP-T cannot increase intracellular ROS levels. The cells in the control group still had no green fluorescence after 520 nm laser irradiation, indicating that light alone did not increase ROS levels. However, the cells incubated with IrP and IrP-T showed obvious green fluorescence, which proved that IrP and IrP-T could produce a large amount of ROS in the cells under light stimulation. Furthermore, the quantitative analysis of fluorescence intensity showed that fluorescence was only present in the presence of IrP and light simultaneously (Figure S7). The difference in fluorescence intensity between IrP and IrP-T cells incubated with light was not significant, indicating that TH287 does not affect ROS production in cells, which is consistent with the previous results of photodynamic experiments. The transient increase in intracellular ROS levels can cause a redox imbalance in cancer cells, leading to oxidative stress, oxidative damage, and cell death.

Avidin readily binds to biotin, while 8-oxo-dGTP possesses a similar structure to biotin, and thus 8-oxo-dGTP can be specifically labeled with avidin [25,37]. When 8-oxo-dGTP is labeled with avidin, the fluorescent group Cy3 attached to avidin can be excited to produce green light. As shown in Figure 3h, cells incubated with IrP-T showed green fluorescence without light irradiation, because TH287 released by IrP-T increased the level of 8-oxo-dGTP in the cells. Under light irradiation, cells incubated with IrP and IrP-T exhibited green fluorescence due to elevated intracellular ROS levels, which increased oxidized 8-oxo-dGTP as well. In addition, compared with the control group, the fluorescence intensities of the IrP + L, IrP-T, and IrP-T + L groups were 4.3, 5.6, and 7.7 times higher, respectively (Figure S8). The fluorescence of the IrP-T + L group was the highest, manifesting that the ROS produced by IrP-T in cells and the released TH287 could effectively synergistically magnify the expression of 8-oxo-dGTP.

According to the above results, when IrP-T is taken up by 4T1 cells, IrP-T produces ROS under light stimulation, and the large amount of toxic ROS produced in the cells leads to the increase in the 8-oxo-dGTP expression level. After photoexcitation, the IrP-T sensitizer synergistically combines PDT with intracellular oxidative stress to enhance the effect of PDT.

### 2.3. In Vivo Anti-Tumor Study

Before assessing the anti-tumor effect of IrP-T in vivo, the distribution of IrP-T in tumor-bearing mice after tail vein injection was investigated. At 6, 12, and 24 h after tail vein injection, the fluorescence of IrP was collected by in vivo small animal imaging to determine the distribution and enrichment in mice. As shown in Figure S9, the red circle marked the tumor region. There was no fluorescence at the tumor site after the 6 h injection. The fluorescence was significantly enhanced at 12 h post-injection, while high-intensity fluorescence appeared at 24 h, indicating that IrP-T was continuously enriched in the tumor over time. The change in fluorescence in tumor-bearing mice suggests that IrP-T can target tumors via the enhanced permeability and retention (EPR) effect. This is because the looser blood vessel walls in the tumor area facilitate nanoparticles to penetrate the tumor more easily. Meanwhile, the lymphatic reflux in the tumor area is blocked, and the tissue blood does not circulate, allowing nanoparticles to remain in the tumor tissue for a long time.

**Figure 4:**
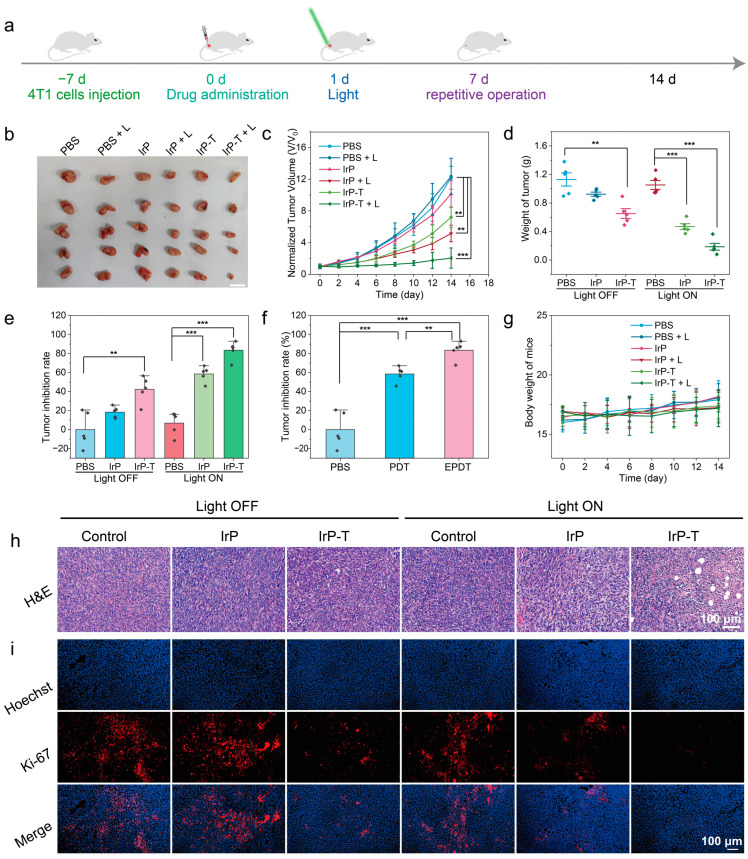
Anti-tumor study of IrP-T in vivo. (**a**) Schematic diagram of IrP-T therapeutic strategy; (**b**) Ex vivo tumor photos of mice after treatment (Scale bar: 2 cm); Mice after 14 days of different treatments tumor volume ratio (**c**), tumor weight (**d**), and tumor inhibition rate (**e**); (**f**) In vivo evaluation of the control group, PDT group (IrP + L), and EPDT group (IrP-T + L); (**g**) The weight change curve of mice treated with different treatments within 14 days; (**h**) H&E staining; (**i**) Ki-67 immunofluorescence staining. (** *p* < 0.01, and *** *p* < 0.001).

The EPDT effect in mice was further investigated based on the respectable EPDT effect and the passive tumor targeting ability of the IrP-T sensitizer at the cellular level. According to the results of the cell level and in vivo fluorescence imaging, a corresponding therapy strategy was designed (Figure 4a). The tumor-bearing mice were randomly divided into six groups (n = 5): PBS, IrP, and IrP-T, with or without light stimulation. IrP-T was injected into mice via the tail vein, light irradiation was started 24 h later, and mice were treated twice over a 14-day cycle.

The tumor volume of the PBS group increased rapidly within 14 days under both light and no-light conditions, illustrating that light has no inhibitory effect on the tumor. In the absence of light, tumor growth in the IrP group was comparable to that in the PBS group, indicating that IrP was not toxic. Tumor growth was considerably reduced in the IrP-T group because the TH287 promoted intracellular oxidative stress, resulting in the blocking of oxidative damage repair and the inhibition of tumor growth. In the presence of light, the tumor volume in the IrP group increased sluggishly and was markedly smaller than that in the PBS group, due to the production of ^1^O_2_ in the tumor. Notably, the tumors in the IrP-T group showed almost no growth, indicating that the produced ^1^O_2_ inhibited tumor cell growth synergistically with TH287 (Figure 4b,c). Furthermore, the weight of isolated tumors in the six groups differed noticeably, with the IrP-T + L group showing the greatest tumor inhibition effect (Figure 4d). Subsequently, the tumor suppression rate was further investigated. Compared with the PBS group, the tumor suppression rates of the IrP + L group, the IrP-T group, and the IrP-T + L group were 58.51%, 42.36%, and 83.51%, respectively (Figure 4e). Additionally, quantitative analysis revealed that the EPDT group had the best therapeutic effect, demonstrating that the organic synergy between PDT and oxidative damage repair blocking could be achieved in solid tumors as well (Figure 4f). Furthermore, the weight of the tumor-bearing mice increased slightly throughout the treatment cycle (Figure 4g), demonstrating that the treatment process had little effect on the lives of tumor-bearing mice.

H&E staining and Ki-67 immunofluorescence staining were used to further evaluate the damage caused by EPDT. Figure 4h shows that the cell morphology of the control group is complete and the nucleus is round, while the tumor cell morphology of the IrP-T + L group is severely damaged, and the nucleus is shrunk or even broken. Ki-67 was then used to verify whether the therapy had stopped tumor cell proliferation (Figure 4i). The results of section staining showed that the cells in the control group had gobs of red fluorescence, whereas the cells in the IrP-T + L group had the least, stressing that the cell proliferation ability was inhibited. The anti-tumor effect in vivo and the tumor section staining experiments both proved that the IrP-T sensitizer had a respectable EPDT effect in solid tumors.

### 2.4. Biosafety Evaluation

**Figure 5:**
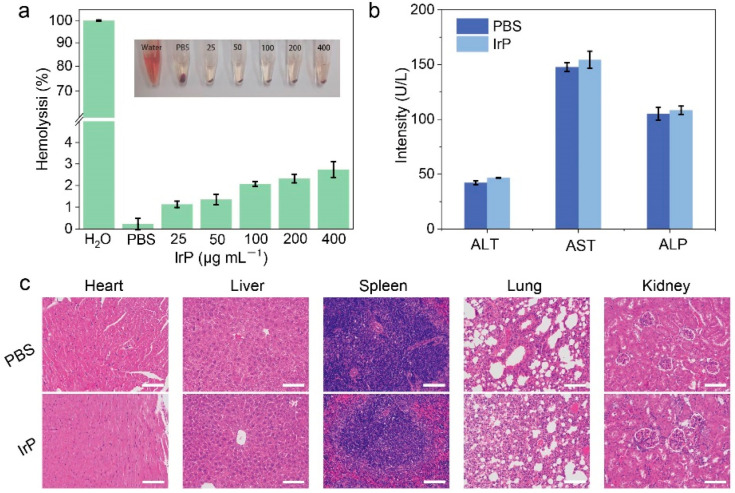
Biocompatibility evaluation of IrP. (**a**) Analysis of hemolysis rate of different concentrations of IrP; (**b**) Liver function analysis (*n* = 3); (**c**) H&E staining of major organs of mice 14 days after tail vein injection of PBS and IrP (80 μg mL^−1^). (Scale bar: 100 μm).

Moreover, the blood routine indexes of mice were studied further. There were no obvious differences in the blood indexes of mice injected with IrP compared with mice injected with PBS (Table S1). In vitro hemolysis experiment results found that the hemolysis rate increased from 1.13% to 2.74% as the IrP concentration increased, which was less than the crucial secure hemolytic ratio for biomaterials (5%), as established by ISO/TR 7406 (Figure 5a). The liver and renal function indexes of mice in both groups did not change greatly after intravenous injection of IrP and PBS (Figure 5b), commenting that IrP did not cause obvious toxicity to mice during the treatment cycle. H&E staining of the main organs (heart, liver, spleen, lung, and kidney) revealed no significant inflammation or tissue destruction (Figure 5c). These findings suggest that IrP has agreeable biocompatibility as a self-assembled nanocarrier and a promising future as a drug delivery system.

## 3. Materials and Methods

### 3.1. Synthesis and Characterization of IrP

The 2,3-naphthalenediamine (0.500 g, 3.2 mmol) and the 1,2-diphenyl ethanedione (0.664 g, 3.2 mmol) were dissolved in 15 mL C_2_H_5_OH. Then, the mixture was stirred and heated at 80 °C for 5 h. Then, the mixture was cooled and crystalized in an ice–water mixture for 1 h. After filtering, the solid was obtained and washed with a small amount of C_2_H_5_OH. After freeze-drying, 0.99 g green crystal powder (85% yield) was obtained. Ir1 (334.5 mg, 1.0 mmol) and IrCl_3_ (150.0 mg, 0.5 mmol) were added to the mixed solvent of 2-ethoxyethanol and deionized water (20 mL, 3:1, *v/v*). The mixture was refluxed under argon protection for 24 h. When the reaction was complete, the solid was harvested by centrifugation. Brownish solid (643.7 mg, 72% yield) was obtained after freeze-drying.

Ir2 (161.5 mg, 0.1 mmol) and Phen-PEG (396.8 mg, 0.18 mmol) were dissolved in mixed solvents of CH_3_OH and CH_2_Cl_2_ (15 mL, 1:2, *v/v*). Then, F_3_CSO_3_Ag (46 mg, 0.18 mmol) was added. The mixture was heated and refluxed at 60 °C for 24 h under argon protection. When the reaction was complete, the solvent was cooled down to room temperature. Then, NH_4_PF_6_ (220.8 mg, 1.4 mmol) was added and the mixture was further stirred for 30 min. After the solvent was removed, the residue was purified by column chromatography (a silica gel), with mixed CH_3_OH/CH_2_Cl_2_ (1:25, *v/v*) as eluent to afford a brown–red solid 220.8 mg (40% yield). 

### 3.2. Photophysical Properties of IrP

The mass extinction coefficient of IrP in dichloromethane solution was determined by a spectrophotometer (Shimadzu UV-1900, Kyoto, Japan). The absorption spectra of IrP (70 μg mL^−1^) were also measured after IrP was dissolved in different solvents including dichloromethane, dimethyl sulfoxide, ethanol, acetonitrile, water, and phosphate buffer saline (PBS, pH = 7.4). The emission spectra of IrP and IrP-T solutions were collected by a spectrometer (NOVA2S-EX, Ideoptics, Shanghai, China) under 520 nm laser irradiation.

### 3.3. IrP Loading TH287

TH287 (600 μM) was dissolved in 0.2 mL dimethyl sulfoxide to form a solution, and then the mixture was slowly added to IrP aqueous solution (320 μg mL^−1^, 3.8 mL) under strong stirring. After 30 min, IrP-T nanomicelles were formed. The self-assembly of IrP is the same as above; IrP dimethyl sulfoxide solution (0.2 mL, 320 μg mL^−1^) was dropped into 3.8 mL deionized water and strongly stirred for 30 min.

### 3.4. Characterization of IrP and IrP-T

The morphology of IrP and IrP-T was observed by transmission electron microscopy (TEM, Hitachi HT7800, Kyoto, Japan). The hydrated particle size (DLS) and polymer dispersion indexes (PDI) of IrP and IrP-T nanomicelles in an aqueous solution for seven days were measured by a size analyzer (Malvern Zetasizer Nano ZS90, Great Malvern, UK).

### 3.5. Singlet Oxygen Detection

The singlet oxygen emission of IrP (2 mL) was measured by a fluorescence spectrometer (Edinburgh FS5, Edinburgh, UK) under 420 nm excitation. The ROS capture agent DPBF was used to determine the yield of ^1^O_2_. The absorption spectrum of IrP aqueous solution containing DPBF (100 μM) was measured under 520 nm light irradiation with time, and the absorbance at 415 nm was recorded. According to the experimental values, the singlet oxygen yields of IrP and IrP-T were calculated by using the formula: Φ_Sam_ = Φ_Ref_ × W_sam_/W_Ref_ × I_Ref_/I_Sam_ × η_Sam_/η_Ref_, where W is the degradation rate constant of DPBF obtained by calculation; I is the absorbance of the sample at 520 nm, I = 1−10^−OD^, OD is the absorbance of the sample at 520 nm; and η is the refractive index of the solvent [38]. The singlet oxygen yields of IrP and IrP-T under 100 mW cm^−2^ laser irradiation were calculated.

### 3.6. Cellular Uptake

Mouse breast cancer (4T1) cells were purchased from the cell bank of the Chinese Academy of Sciences; mouse breast cancer cells were cultured in DMEM medium (containing 10% FBS and 1% penicillin-streptomycin) in a BB150 carbon dioxide incubator (37 °C, 5% CO_2_). IrP-P nanomicelles were prepared by replacing TH287 with red fluorescence dye (protoporphyrin IX, PpIX, hereinafter P). 4T1 cells were seeded in cell culture dishes for 24 h, and then IrP-P DMEM solution (P = 10 μM, 1.0 mL) was incubated with cells for 0.5, 1, 2, 4, and 8 h. After washing with PBS, the cells were observed under a confocal fluorescence microscope (Zeiss LSM900, Germany, λ_ex_ = 488 nm, λ_em_ = 632 nm).

### 3.7. Cell Viability Detection

Cell counting kit-8 (CCK-8) was used to evaluate cell viability [39]. Normally, 4T1 cells were digested with trypsin and then seeded in 96-well plates at a density of 1 × 10^4^ cells per well. After 12 h, the culture medium was replaced with IrP and IrP-T Dulbecco’s modified eagle medium (DMEM) solution. The cells were further incubated for 4 h and then washed with fresh DMEM. The cells were irradiated with 520 nm light (100 mW cm^−2^) for 5 min and then incubated for 20 h. After washing with PBS, CCK-8 was added and incubated for 40 min. The absorbance (Abs.) at 450 nm was measured by a microplate reader, and then the cell viability was calculated using the following formula [40]: Cell viability (%) = (Abs.sample − Abs.blank)/(Abs.control − Abs.blank) × 100%

### 3.8. Live/Dead Cell Staining

4T1 cells were stained with live/dead cells kits (Calcein-AM and PI), respectively. 4T1 cells were seeded in 24-well plates and cultured for 12 h. Subsequently, the cells were incubated with PBS, IrP, and IrP-T DMEM solution (IrP = 16 μg mL^–1^; TH287, T = 60 μM) for 4 h, and then irradiated with a 520 nm laser (100 mW cm^−2^) for 5 min. After 20 h, the Calcein-AM/PI solution was incubated with cells for 20 min. The cells were washed three times with PBS, and then the green fluorescence of Calcein-AM and the red fluorescence of PI were collected by a fluorescence microscope and photographed.

### 3.9. Intracellular ROS Detection

A 2′,7′-Dichlorodihydrofluorescein diacetate (DCFH-DA) probe was used to detect intracellular ROS. 4T1 cells were seeded in 24-well plates and cultured for 12 h. Subsequently, 4T1 cells were treated with PBS, IrP, and IrP-T DMEM solution (IrP = 16 μg mL^−1^; TH287, T = 60 μM) for 4 h, and then irradiated with a 520 nm laser (100 mW cm^−2^) for 5 min. After 2 h, the cells were washed with PBS and cultured with DCFH-DA DMEM solution for 10 min. After washing with PBS, the green fluorescence of DCF was collected by a fluorescence microscope and photographed.

### 3.10. Immunofluorescence

Firstly, cells were treated with the above conditions (PBS, IrP, and IrP-T DMEM solution and 520 nm laser irradiation). After 20 h, 4T1 cells were washed with PBS and fixed with 4% paraformaldehyde for 30 min. Next, cells were permeabilized with PBS for 15 min and blocked with 15% FBS for 2 h. Subsequently, cells were incubated with Avidin-Cy3 antibody (Rockland, A003-04) diluents at 4 °C for 12 h and then incubated with secondary antibodies at 37 °C for 1 h. The nuclei were stained with Hoechst-containing DMEM for 10 min. Signals were collected and photographed using a fluorescence microscope.

### 3.11. Tumor Model

Female BALA/c mice (4 weeks old) were purchased from Shanghai Laboratory Animal Research Center and cultured in a clean environment. The laboratory is authorized by the Shanghai Municipal Commission of Science and Technology (SYXK 2020-0006). All animal-related experiments were performed in the Shanghai Ruitaimosi Biotechnology Co., Ltd. And authorized by the Shanghai Science and Technology Committee (SYXK 2021-0007). 4T1 cells (2 × 10^6^) were subcutaneously injected into the left armpit of mice. When the tumor volume grew to about 100 mm^3^ or so, it was used for the in vivo experiment.

### 3.12. In Vivo Fluorescence Imaging

IrP-T (200 μL, IrP = 80 μg mL^–1^; T = 300 μM) was injected into BALB/c tumor-bearing mice via the tail vein. In vivo imaging of tumor-bearing mice was performed using a small animal in vivo imager at 6, 12, and 24 h after injection. Subsequently, fluorescence images of mice were captured by an in vivo fluorescence imager (λ_ex_ = 730 nm, λ_em_ = 830 nm, IVIS Lumina LT series III, PerkinElmer, USA).

### 3.13. Therapeutic Effect Evaluation of Tumor-Bearing Mice

4T1 tumor-bearing mice were randomly divided into six groups (n = 5). (1) Control group, (2) Light (hereinafter L) group, (3) IrP group, (4) IrP + L group, (5) IrP-T group, and (6) IrP-T + L group. For groups (1) and (2), saline (200 μL) was injected via the tail vein. For groups (3) and (4), IrP (200 μL, IrP = 80 μg mL^–1^) was injected via the tail vein. For groups (5) and (6), IrP-T (200 μL, IrP = 80 μg mL^–1^; T = 300 μM) was injected via the tail vein. Twenty-four hours after injection, groups (2), (4), and (6) were illuminated with a 520 nm laser (100 mW cm^−2^) for 5 min. During the 14-day treatment cycle, the treatment was repeated on day 7. The body weight and tumor volume of the mice were measured every two days. After 14 days, the tumors of the mice were removed, photographed, and weighed. The relative volume of the tumor was calculated by the following formula: Volume = Length × width^2^/2

### 3.14. H&E Staining and Immunofluorescence Staining

The tumors of the treated tumor-bearing mice were removed and cut into sections, and H&E staining experiments and immunofluorescence experiments (Ki-67) were performed. Tumors were fixed in a 4% formaldehyde solution for 24 h, embedded in paraffin, and then sliced into sections with a thickness of 5 µm. Subsequently, the sections were stained with H&E and Ki-67 antibody (Cell Signaling Technology, D3B5). The major organs (heart, liver, spleen, lung, and kidneys) were also collected for H&E staining to evaluate the toxicity and side effects of treatment in vivo, and then the stained sections were captured by an optical microscope.

### 3.15. Statistical Analysis

A two-tailed Student’s *t*-test was performed for the comparison of two groups. For multiple comparisons, a one-way analysis of variance (ANOVA) test was performed. Statistical significance was set at *p* < 0.05 (* *p* < 0.05, ** *p* < 0.01, *** *p* < 0.001).

## 4. Conclusions

In this study, we developed an iridium complex-based drug-loaded nanoplatform for the EPDT of tumors. IrP, an amphiphilic iridium complex, was obtained by modifying polyethylene glycol on the N^N ligand and then coordinating an iridium dimer with the N^N ligand. IrP exhibits excellent photophysical and photochemical properties. It absorbs light well at 520 nm and emits long-wavelength red light, making it a potential for tumor imaging and treatment. The amphiphilic IrP loaded TH287 to form IrP-T nanomicelles by self-assembly. The average particle size of IrP-T is 260 nm and can be stably maintained in water for a long time. The ^1^O_2_ yield of IrP-T is 0.36, indicating that it has a vigorous ability to produce ^1^O_2_. Cell-level experiments revealed that IrP-T can improve the inhibition of cell viability by the EPDT effect. Mechanistic studies have shown that TH287 can synergistically boost intracellular oxidative stress, and achieve tumor cell death. Anti-tumor experiments in vivo also proved that IrP-T can achieve a satisfactory EPDT effect. Furthermore, blood routine and organ H&E staining demonstrated that IrP was safe and had potential as a drug delivery platform. Therefore, the combination strategy of light-stimulated ROS generation and blocking oxidative damage repairing offers a novel strategy for the oxygen-saved anti-tumor proliferation treatment.

## Data Availability

Not applicable.

## Supplementary Materials

The following supporting information can be downloaded at: https://www.mdpi.com/article/10.3390/molecules28114397/s1, Scheme S1: Synthesis route of IrP; Figure S1: (a) ^1^H-NMR spectrum and (b) high-resolution mass spectrum of IrP; Figure S2. Absorption curves of IrP-T (IrP = 16 μg mL^−1^, different concentrations of TH287); Figure S3: Degradation normalization curves of DPBF (a) IrP with different concentrations (100 mW cm^−2^) and (b) different light power densities (IrP = 32 μg mL^−1^); Figure S4: Absorption spectra (a) and normalized absorption at 260 nm with time (b) of the mixture of NBT and IrP at 260 nm under 520 nm light irradiation; Figure S5: Statistical particle size distribution of IrP (a) and IrP-T (b); Figure S6: Cell viability fitting curve and IC_50_ value of IrP + L group; Figure S7: Fluorescence intensity histogram of intracellular ROS after different treatments; Figure S8. Fluorescence intensity histogram of intracellular 8-oxo-dGTP after different treatments; Figure S9: In vivo fluorescence imaging of mice 6 h, 12 h, and 24 h after tail vein injection of IrP-T (λ_ex_ = 730 nm, λ_em_ = 830 nm); Table S1: Blood routine indexes of mice.

## Sample Availability

Samples of the compounds are not available from the authors.
