# Supplementary material for: Light-Elicited and Oxygen-Saved Iridium Nanocapsule for Oxidative Damage Intensified Oncotherapy
Source: Molecules. 2023 May 28;28(11):4397. doi: 10.3390/molecules28114397 (PMC10254276; doi:10.3390/molecules28114397)
Supplement: Supplementary file 1 [file molecules-28-04397-s001.zip › molecules-2381417-supplementary.pdf]

Supporting information

Light-elicited and oxygen-saved iridium nanocapsule for oxidative damage intensified oncotherapy

Guobo Chen[1,†], Xiang Wang[1,†], Zongyan He[1], Xueyu Li[1], Zhijin Yang[2], Yule Zhang[2], Yuhao Li[1,]*, Lulu Zheng[2,]*, Yuqing Miao[1], and Dawei Zhang[2]

[1] School of Materials and Chemistry, Institute of Bismuth, University of Shanghai for Science and Technology, Shanghai 200093, China.

[2] Shanghai Environmental Biosafety Instruments and Equipment Engineering Technology Research Center, Engineering Research Center of Optical Instrument and System, the Ministry of Education & Shanghai Key Laboratory of Modern Optical System, University of Shanghai for Science and Technology, Shanghai 200093, China.

† Guobo Chen and Xiang Wang contributed equally

* Corresponding author:

Yuhao Li (Email: yhli@usst.edu.cn)

Lulu Zheng (llzheng@usst.edu.cn)

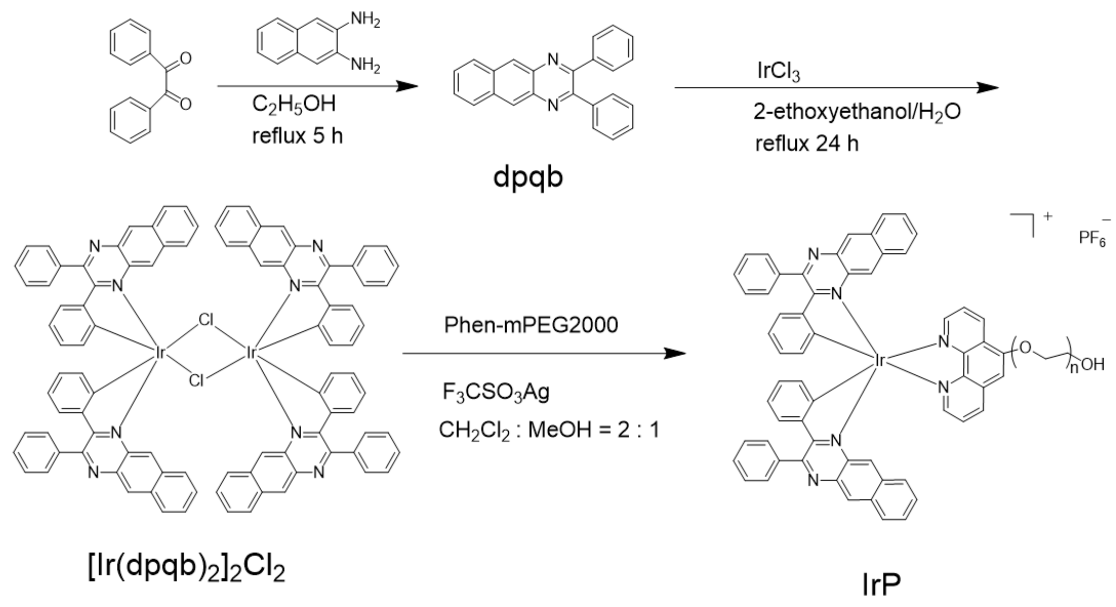

Scheme S1. Synthesis route of IrP.

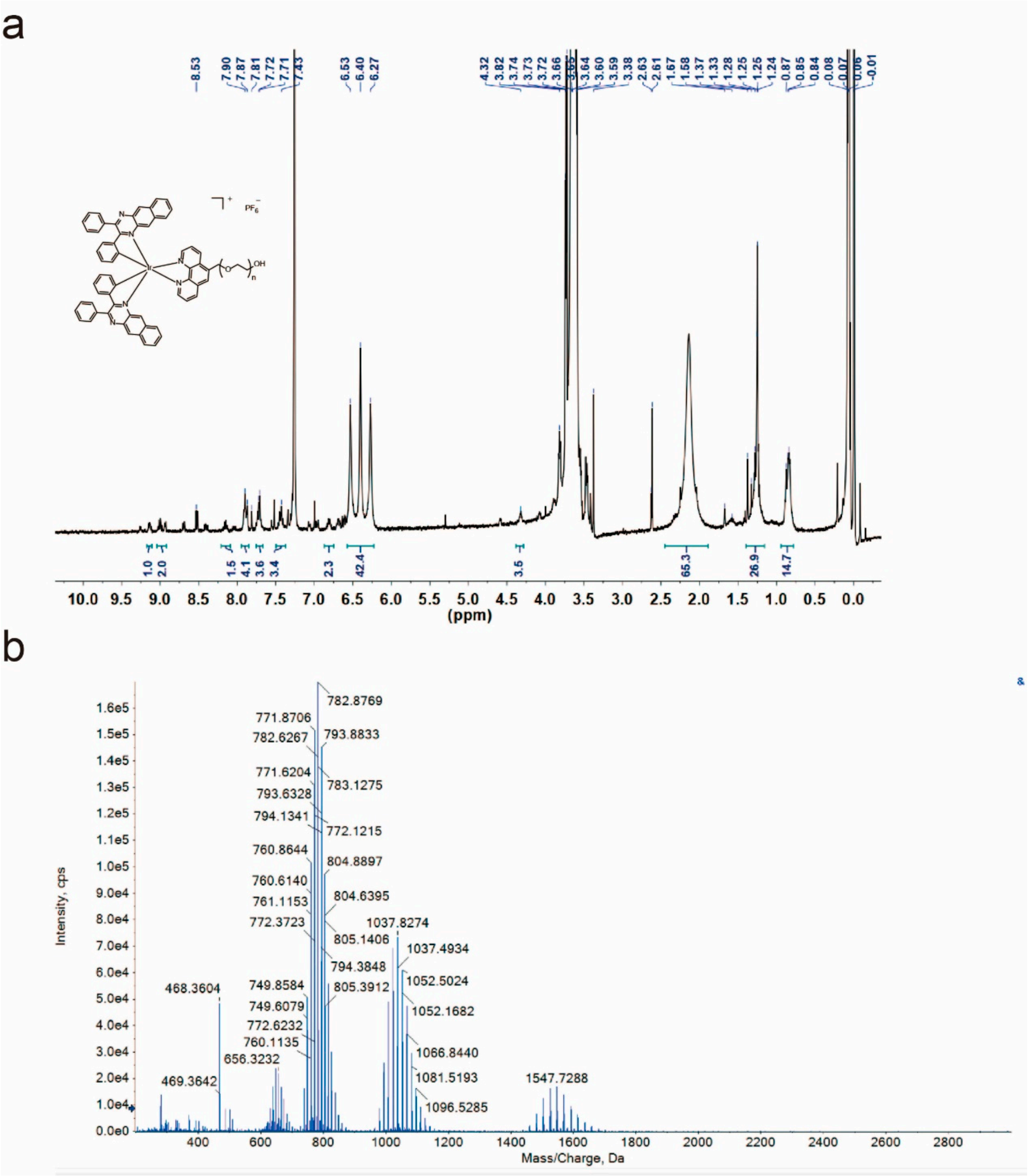

Figure S1. (a) ^{1}H-NMR spectrum and (b) high-resolution mass spectrum of IrP.

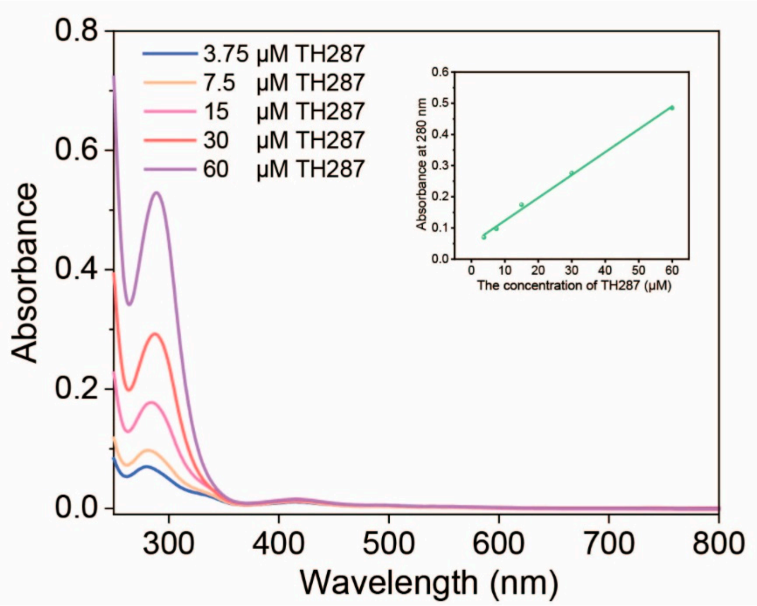

Figure S2. Absorption curves of IrP-T (IrP = 16 μg mL^{-1}, different concentrations of TH287).

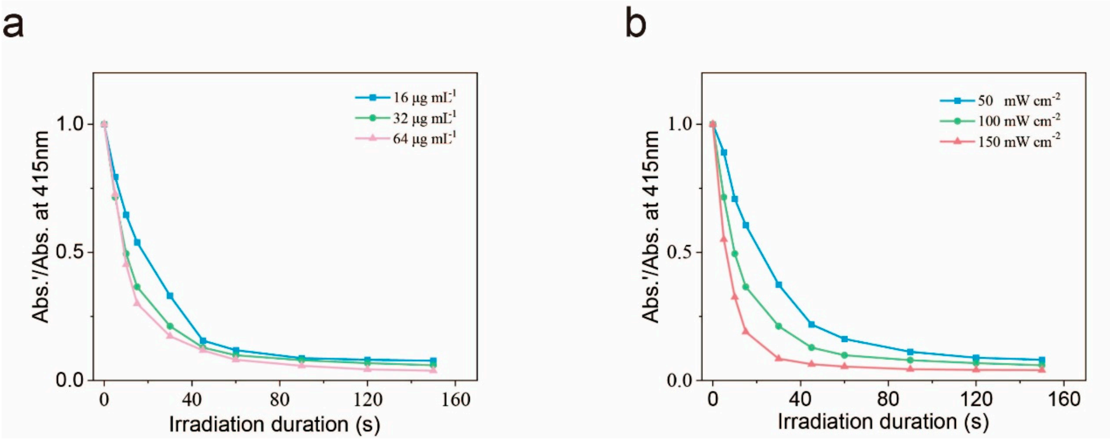

Figure S3. Degradation normalization curves of DPBF (a) IrP with different concentrations (100 mW cm^{-2}) and (b) different light power densities (IrP = 32 μg mL^{-1}).

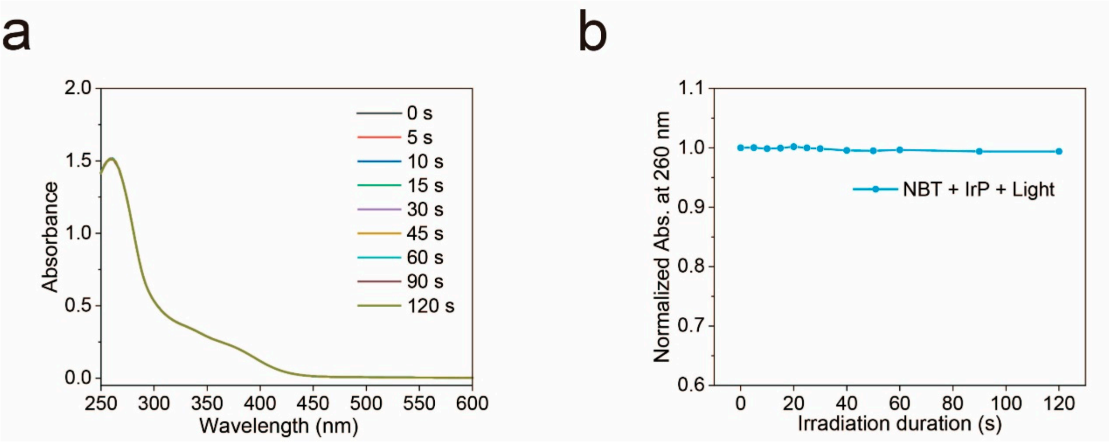

Figure S4. Absorption spectra (a) and normalized absorption at 260 nm with time (b) of the mixture of NBT and IrP at 260 nm under 520 nm light irradiation.

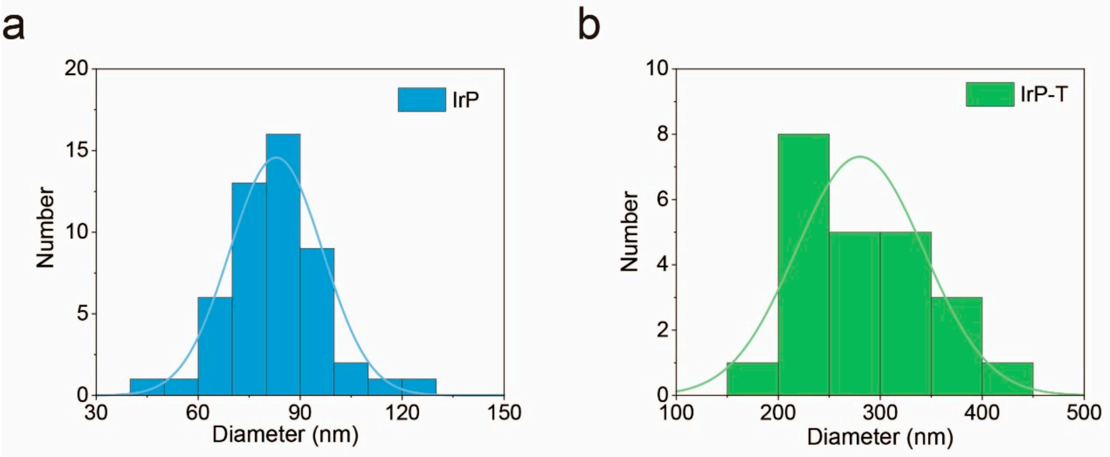

Figure S5. Statistical particle size distribution of IrP (a) and IrP-T (b).

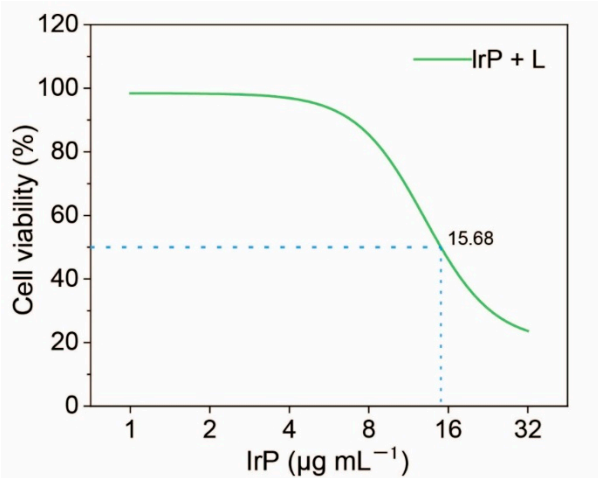

Figure S6. Cell viability fitting curve and IC_{50} value of IrP + L group.

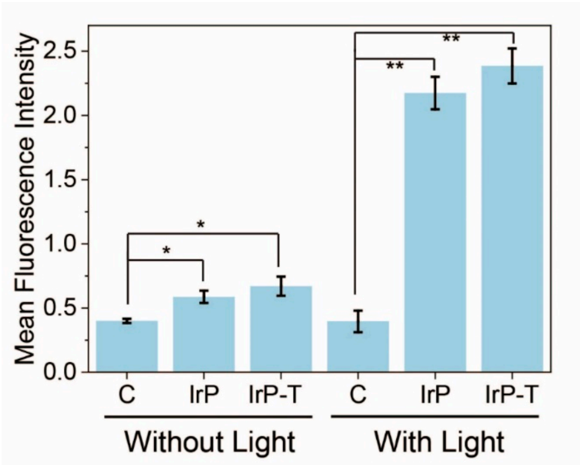

Figure S7. Fluorescence intensity histogram of intracellular ROS after different treatments.

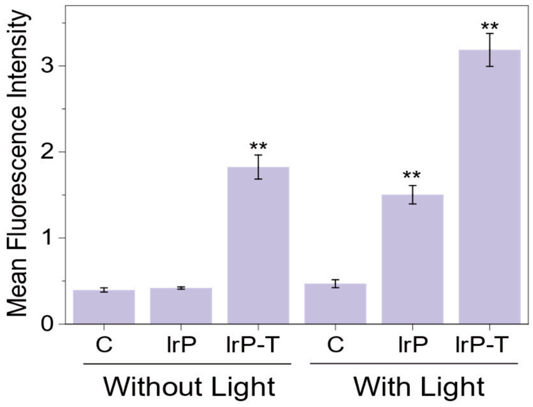

Figure S8. Fluorescence intensity histogram of intracellular 8-oxo-dGTP after different treatments.

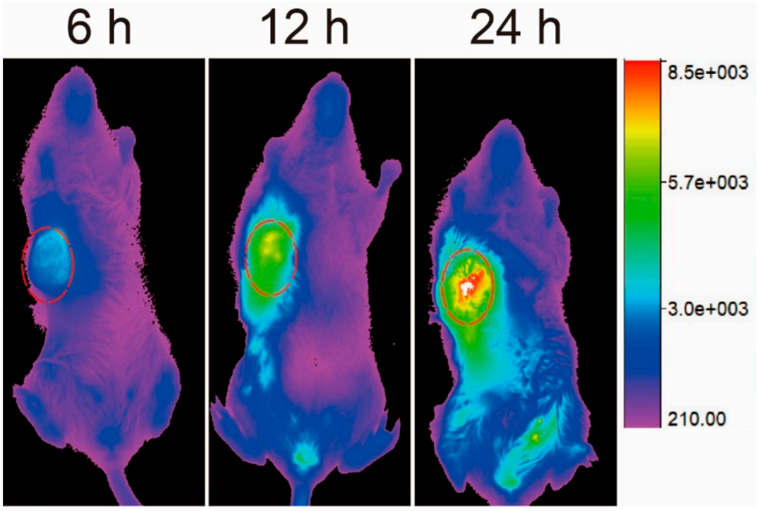

Figure S9. In vivo fluorescence imaging of mice 6 h, 12 h and 24 h after tail vein injection of IrP-T (λ_{ex} = 730 nm, λ_{em} = 830 nm).

Table S1. Blood routine indexes of mice

Index[a]	PBS	IrP
BUN	7.90 ± 0.57	7.10 ± 0.85
UA	157.75 ± 7.99	160.95 ± 11.38
SCR	30.65 ± 0.64	27.50 ± 1.56
Lym	84.95 ± 8.56	74.10 ± 5.66
RBC （10^{12} L^{-1}）	7.72 ± 0.34	8.71 ± 0.07
HCT （%）	41.30 ± 2.12	46.60 ± 0.42
MCV （fL）	53.50 ± 0.42	53.50 ± 0.03
MCH （pg）	17.25 ± 0.21	16.55 ± 0.35
MCHC （g L^{-1}）	322.50 ± 6.36	309.50 ± 6.36
RDW-CV （%）	18.90 ± 0.57	18.85 ± 0.07
RDW-SD （fL）	36.60 ± 0.57	37.00 ± 0.14
PLT （10^9 L^{-1}）	1192.00 ± 263.04	1121.00 ± 66.47
MPV （fL）	7.25 ± 0.07	7.35 ± 0.21
PDW	15.25 ± 0.07	15.30 ± 0.11

[a] BUN：blood urea nitrogen；UA：Uric Acid；SCR：Serum creatinine；Lym：lymphocyte；RBC：red blood cell；HGB：hemoglobin；HCT：hematocrit；MCV：mean corpusular volume；MCH：mean corpusular hemoglobin；MCHC：mean corpuscular hemoglobin concentration；RDW-CV：Red cell distribution width coefficient of variation；RDW-SD：Red blood cell distribution width SD；PLT：platelet count；MPV：mean platelet volume；RDW：Red blood cell distribution width.
